# New Zealand Sujon blackcurrant lowers lactate accumulation during cycling in triathletes

**DOI:** 10.1186/1550-2783-11-S1-P2

**Published:** 2014-12-01

**Authors:** Mark ET Willems, Stephen D Myers, Mandy L Gault, Matthew D Cook

**Affiliations:** 1 Department of Sport and Exercise Sciences, University of Chichester, Chichester, United Kingdom

## Background

Blackcurrant intake has been reported to increase peripheral blood flow in humans [1], potentially by anthocyanin-induced vasorelaxation and vasodilation [2]. Increased peripheral blood flow may affect the exercise intensity at lactate indicators (e.g. onset of blood lactate accumulation (OBLA) at 4 mmol L^-1^) and maximum oxygen uptake. We examined the effect of 1-week Sujon blackcurrant powder supplementation on the blood lactate curve and aerobic capacity of trained triathletes.

## Methods

Healthy male (n=8) and female (n=5) triathletes with >3 yrs experience (age: 38±8 yrs, height: 174±5 cm, body mass: 71±9 kg, BMI: 23±2, BF%: 19±5%, mean±SD) performed cycling tests for lactate responses (4 min stages with 2 min recovery, start power 50 W with 30 W increments) and maximum oxygen uptake (start power 50 W for 4 min with 30 W min^-1^ increments) at self-selected pedal cadence (SRM ergometer, SRM International, Germany). Familiarized participants were tested following 7 days of Sujon blackcurrant powder (S, 6g/day) or placebo (P) intake. Experimental design was double-blind and randomized with a wash-out period of 4 weeks. Oxygen consumption (Douglas bag technique) and heart rate were recorded during the cycling tests. Intensity, oxygen uptake and heart rate at 4 mmol L^-1^ OBLA were calculated using lactate analysis software (Newell et al., 2007). Lactate responses were calculated at relative intensities with individual lactate curves. Paired t-tests were used for analysis with significance accepted at p<0.05. Consent to publish the results was obtained from all participants.

## Results

The intensity at 4 mmol L^-1^ OBLA was 6% higher with Sujon (P: 223±57, S: 236±60 W, range -5 to 22%, 11 participants showed an increase and 1 no change). In both conditions at 4 mmol L^-1^ OBLA, there were no differences in heart rate (P: 159±7, S: 164±10 b min^-1^, p=.13) or oxygen uptake (P: 2.91±0.73, S: 2.96±0.71 L min^-1^, p=.31). Blood lactate was lower at 40% (P: 1.24±0.52, S: 0.91±0.46 mmol L^-1^), 50% (P: 1.58±0.78, S: 1.23±0.64 mmol L^-1^), 60% (P: 2.29±0.96, S: 1.91±0.87 mmol L^-1^) and 70% (P: 3.52±1.10, S: 3.08±1.21 mmol L^-1^) of maximum power, decreases of 27%, 22%, 17% and 13%, respectively. There was no effect on maximum values of oxygen uptake (P: 49.1±6.2, S: 49.7±6.1 mL kg^-1^ min^-1^), power (P: 305±68, S: 307±62 W) or heart rate (P: 172±10, S: 172±11 b min^-1^). However, maximum oxygen uptake with Sujon was obtained with 14% lower lactate values (measured 3-min after exhaustion; P: 7.85±1.69, S: 6.79±1.51 mmol L^-1^, range -27 to 48%, 10 participants showed a decrease and 1 no change).

## Conclusions

Intake of New Zealand Sujon blackcurrant powder is associated with 1) a substantial downward and rightward shift of the lactate curve during cycling over a wide range of intensities, and 2) lower lactate accumulation at aerobic capacity suggesting increased lactate clearance or altered substrate oxidation. These findings may have implications for training practice and aerobic performance of endurance athletes.
